# XIAP Protection of Photoreceptors in Animal Models of Retinitis Pigmentosa

**DOI:** 10.1371/journal.pone.0000314

**Published:** 2007-03-21

**Authors:** Kevin C. Leonard, Dino Petrin, Stuart G. Coupland, Adam N. Baker, Brian C. Leonard, Eric C. LaCasse, William W. Hauswirth, Robert G. Korneluk, Catherine Tsilfidis

**Affiliations:** 1 University of Ottawa Eye Institute, Ottawa, Ontario, Canada; 2 Ottawa Health Research Institute, Ottawa, Ontario, Canada; 3 University of Florida, Gainesville, Florida, United States of America; 4 Apoptosis Research Centre, Children's Hospital of Eastern Ontario, Ottawa, Ontario, Canada; University of Florida, United States of America

## Abstract

**Background:**

Retinitis pigmentosa (RP) is a blinding genetic disorder that is caused by the death of photoreceptors in the outer nuclear layer of the retina. To date, 39 different genetic loci have been associated with the disease, and 28 mutated genes have been identified. Despite the complexity of the underlying genetic basis for RP, the final common pathway is photoreceptor cell death via apoptosis.

**Methodology/Principal Findings:**

In this study, P23H and S334ter rhodopsin transgenic rat models of RP were used to test the neuroprotective effects of anti-apoptotic gene therapy. Adeno-associated viruses (AAV) carrying the X-linked inhibitor of apoptosis (XIAP) or green fluorescent protein (GFP) were delivered subretinally into the eye of transgenic rat pups. Histological and functional measures were used to assess neuroprotection. XIAP is known to block apoptosis by inhibiting the action of caspases-3, -7 and -9. The results show that XIAP gene therapy provides long-term neuroprotection of photoreceptors at both structural and functional levels.

**Conclusions/Significance:**

Our gene therapy strategy targets the apoptotic cascade, which is the final common pathway in all forms of retinitis pigmentosa. This strategy holds great promise for the treatment of RP, as it allows for the broad protection of photoreceptors, regardless of the initial disease causing mutation.

## Introduction

Retinitis pigmentosa (RP) is a genetically heterogeneous group of retinal degenerations and the most common cause of inherited blindness in the developed world, with an estimated incidence between 1 in 3000 to 1 in 5000 [1]. The disease is caused by the degeneration of the photoreceptor cells, with rod photoreceptor death preceding the secondary loss of cones. RP is characterized by progressive night blindness, reduction or loss of visual acuity and constriction and gradual loss of the visual field [2], [3]. To date, 39 different genetic loci have been associated with the disease, and 28 mutated genes have been identified [4]. Within these 39 genes, there are many individual mutations that cause RP. Rhodopsin mutations are the most prevalent, with over 100 identified. Despite the complexity of the underlying genetic basis for RP and other similar retinal degenerative diseases, the final common pathway is photoreceptor cell death via apoptosis [5]–[8].

There are a variety of well established rodent models of RP, based on both spontaneous and engineered genetic mutations. For the current study, the P23H and S334ter rhodopsin transgenic rat models were used. The P23H rat has a histidine substituted for proline in position 23 of the rhodopsin gene [9]. The same mutation is causative in the most prevalent form of autosomal dominant retinitis pigmentosa (adRP) in North America, being linked to approximately 12% of human cases [10]. In the S334ter rats, the rhodopsin gene has a termination codon at residue 334 which results in the expression of rhodopsin proteins which lack the 15 C-terminal amino acids [11]. The C-terminus is involved in rhodopsin trafficking to the photoreceptor outer segments [12] and in the deactivation of the rhodopsin protein after light absorption (reviewed in [13]), both of which are key events in phototransduction. In both rat models, there is a gradual apoptotic loss of photoreceptors in the outer nuclear layer (ONL) of the retina.

The loss of photoreceptors in RP has been shown to occur via apoptosis [7]. Apoptotic pathways most often involve a family of cysteine proteases known as caspases [14], that cleave key cellular targets, ultimately leading to the death of the cell. A family of proteins known as the inhibitors of apoptosis (IAPs) block cell death by interfering with the activity of key caspases. The IAP proteins contain one or more 70–80 amino acid baculoviral IAP repeat (BIR) domains, which have a conserved cysteine and histidine core sequence (Cx_2_Cx_6_Wx_3_Dx_5_Hx_6_C). The BIR domains and their linker regions bind to and suppress caspase activity [15], [16].

IAPs have been shown to block cellular apoptosis induced by a wide variety of triggers [17], [18]. The X-linked inhibitor of apoptosis (XIAP) protein is the best characterized and most potent IAP family member [19]. XIAP contains three BIR domains which, in conjunction with their linker regions, bind to and inhibit caspases 3, 7, and 9 [17]. Additionally, XIAP contains a carboxy terminal RING zinc finger domain that possesses E3 ubiquitin ligase activity. This domain allows XIAP to auto-ubiquitinate and self-degrade under severe apoptotic stress [20], [21] but can also promote the ubiquitination and degradation of caspases [22] and thus suppress apoptosis in conditions of lower apoptotic stress [23].

Gene therapy strategies employing virally-delivered XIAP have been shown to confer resistance to apoptosis in a variety of in vivo cell death models. XIAP promotes neuroprotection in forebrain [24] and retinal [25] ischemia, in the MPTP model of Parkinson's Disease [26], [27], in retinal ganglion cell death induced by axotomy of the optic nerve [28], [29] or increased intraocular pressure [30], and in cisplatin induced ototoxicity [31]. In addition, we have previously shown that XIAP over-expression can protect photoreceptors both structurally and functionally in a chemotoxic animal model of retinal degeneration [32], [33]. However, all of these previous reports of XIAP protection involve acute models of apoptotic stress. The important question remains whether XIAP gene therapy can be efficacious in models of chronic stress caused by genetic mutation.

The present study examines the neuroprotective effects of XIAP gene therapy in genetic models of RP. We hypothesized that AAV mediated delivery of XIAP should provide neuroprotection in these progressive retinal degenerations given its efficacy in acute insult. The data show that XIAP is able to significantly protect photoreceptors at both the structural and functional level in the P23H model of RP, and protects photoreceptor structure in the S334ter model. Therefore, XIAP gene therapy is of potential use in the treatment of human RP.

## Results

**Figure 1:**
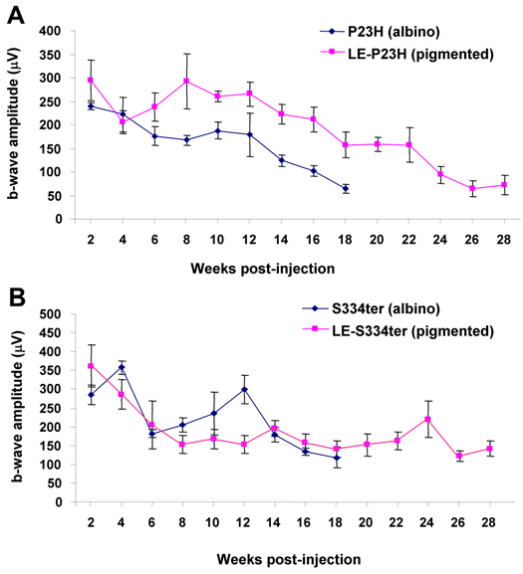
Photoreceptor degeneration is slowed in P23H animals crossed onto the Long Evans (LE) pigmented background in comparison to albino transgenic animals (A), but not in S334ter animals (B). B-wave data (+/- SEM) was taken from the contralateral untreated eyes of experimental animals. N = 5 for all groups.

Homozygous albino P23H and S334ter transgenic animals were crossed onto a pigmented Long Evans background to yield heterozygous pigmented offspring. These crosses were conducted because assessment of neuroprotection in a pigmented eye would more closely resemble the gene therapy situation in humans. Based on electroretinography, the retinal degeneration for these pigmented animals appeared to be unaffected in the S334ter animals, but was somewhat delayed in the P23H animals (Fig. 1A,B), perhaps due to increased phototoxicity for this mutation on the albino background [34]. In order to assess XIAP neuroprotection of photoreceptors in the retinas of pigmented P23H and S334ter animals, rats were given a subretinal injection of hemagglutinin (HA)-tagged AAV-XIAP or AAV-GFP at postnatal day 14–17, when the eyelids opened. Serotype 5 AAV was used to ensure rapid upregulation of the transgene in photoreceptors.

### XIAP Overexpression in the Retina correlates with Structural Protection of Photoreceptor Cells

**Figure 2:**
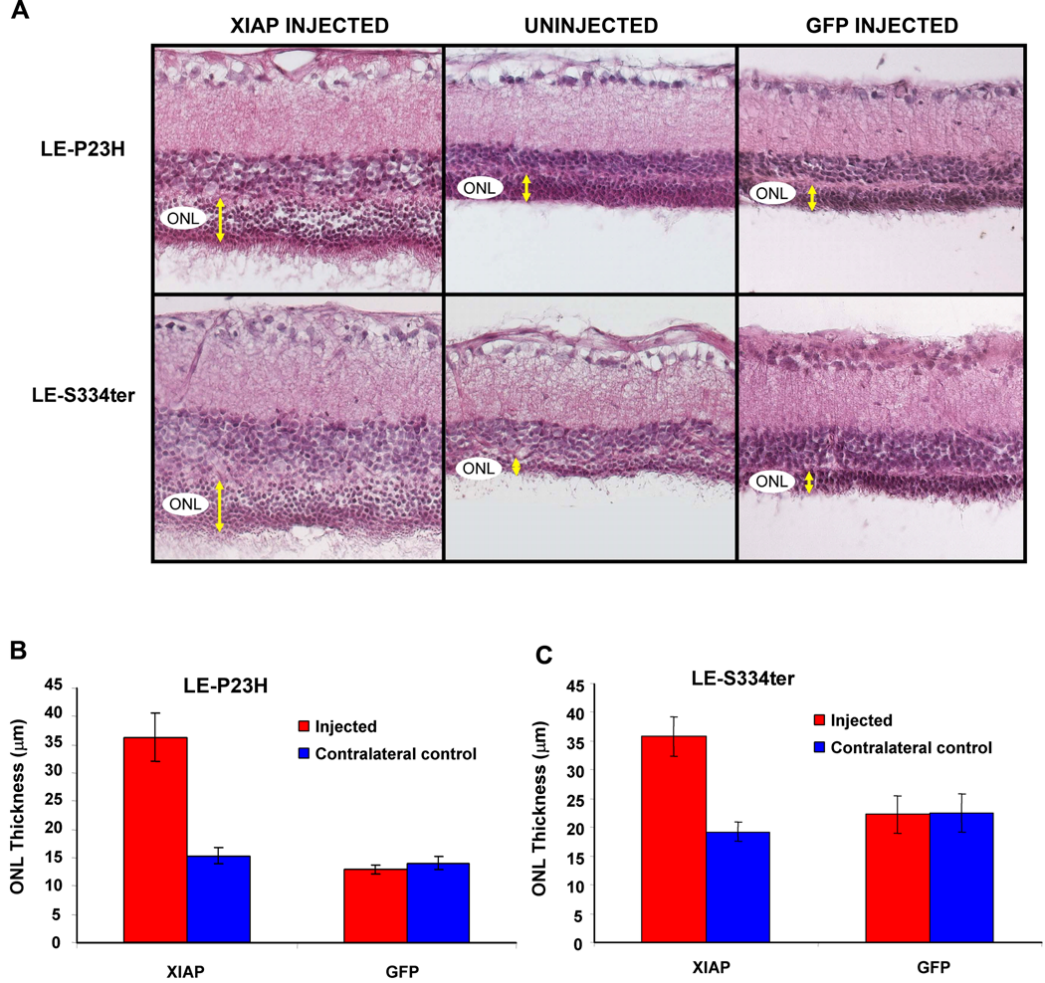
Structural protection of photoreceptors. A) Haematoxylin and Eosin staining of retinal sections at 28 weeks after AAV subretinal injection. XIAP-treated retinas have increased layers of photoreceptor nuclei in comparison to contralateral untreated controls as well as age-matched GFP-treated controls. B) For the LE-P23H animals, XIAP-treatment preserves the ONL in comparison to both the untreated control eyes (p<0.0001) and GFP-treated eyes (p<0.0001) (XIAP N = 16, GFP N = 4). For LE-S334ter, XIAP-treated eyes are similarly significantly better than untreated controls (p<0.001) and GFP-treated eyes (p<0.005) (XIAP N = 15, GFP N = 8).

To assess structural neuroprotection, histology was conducted on eyes sampled at 28 weeks after AAV injection. XIAP treatment preserved outer nuclear layer (ONL) morphology in both P23H and S334ter animals. XIAP-treated retinas possessed multiple layers of photoreceptors (Fig. 2A), and were significantly thicker (Fig. 2B) than their contralateral untreated counterparts as well as similarly age-matched GFP-treated controls.

**Figure 3:**
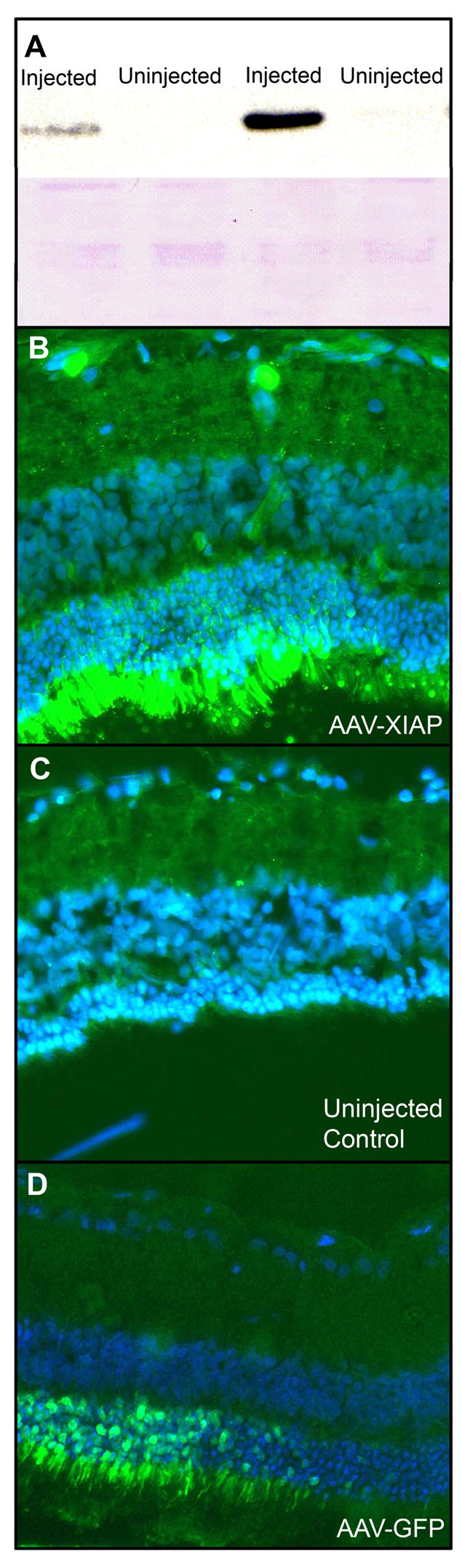
**A**) Western blot confirmation of XIAP over-expression in injected eyes in P23H animals in comparison to the contralateral control uninjected eye in the same animal. Blot was probed with an anti-HA antibody. Given that the protein extracts were made from the whole retina, and only a fraction of the retina was covered by the subretinal injection, XIAP protein levels seen on the Western blot are an under-estimation of the level of over-expression at the site of injection. Some variability in XIAP expression is present between animals. Ponceau red staining of membrane confirms equal loading. (B) Anti-HA immunofluorescence (green) confirms XIAP over-expression at site of neuroprotection in a P23H animal. (C) Contralateral untreated control has no fluorescence and shows diminished ONL thickness. (D) GFP-injected retina of a S334ter animal is shown at boundary between photoreceptors covered by the virus and photoreceptors outside the range of the virus. ONL thickness is similar on either side of the virus boundary. Sections are counterstained with DAPI (nuclear stain).

Western blotting confirmed that the HA-tagged XIAP protein was overexpressed in the retinas of injected eyes and undetectable in the contralateral control eyes (Fig. 3A). Furthermore, immunohistochemistry confirmed XIAP overexpression at sites of photoreceptor preservation (Fig 3B). Using an anti-HA antibody, the tagged XIAP transgene was clearly seen in photoreceptor inner and outer segments and coincided perfectly with preserved ONL morphology. Sections were counterstained with a DAPI nuclear stain in order to emphasize ONL structure and thickness. Moving away from the site of AAV injection, XIAP immunofluorescence was found to diminish, as was ONL thickness. As expected, the retina of the uninjected contralateral eye showed no HA immunofluorescence and possessed a uniformly diminished ONL (Fig. 3C).

Strong GFP fluorescence was observed in the ONL and photoreceptor inner segments of AAV-GFP injected eyes. Retinal sections showing the border between GFP expressing cells and cells not covered by the virus conclusively demonstrated that the thickness of the ONL is unaffected by the presence of AAV-GFP (Fig. 3D).

### Functional Protection of Photoreceptor Cells

Electroretinography was conducted to assess the effects of XIAP gene therapy on retinal function. Electroretinograms (ERGs) record the combined electrical activity of the various components of the retina following flashes of light, and can be divided into a hyperpolarizing a-wave and a depolarizing b-wave. While the b-wave is accepted as a reliable measure of overall retinal function, the a-wave is generated more specifically by the photoreceptor cells. Ratios of the a- and b-wave amplitude in the treated eye to that in the untreated eye were used to assess preservation of visual function following gene therapy.

**Figure 4:**
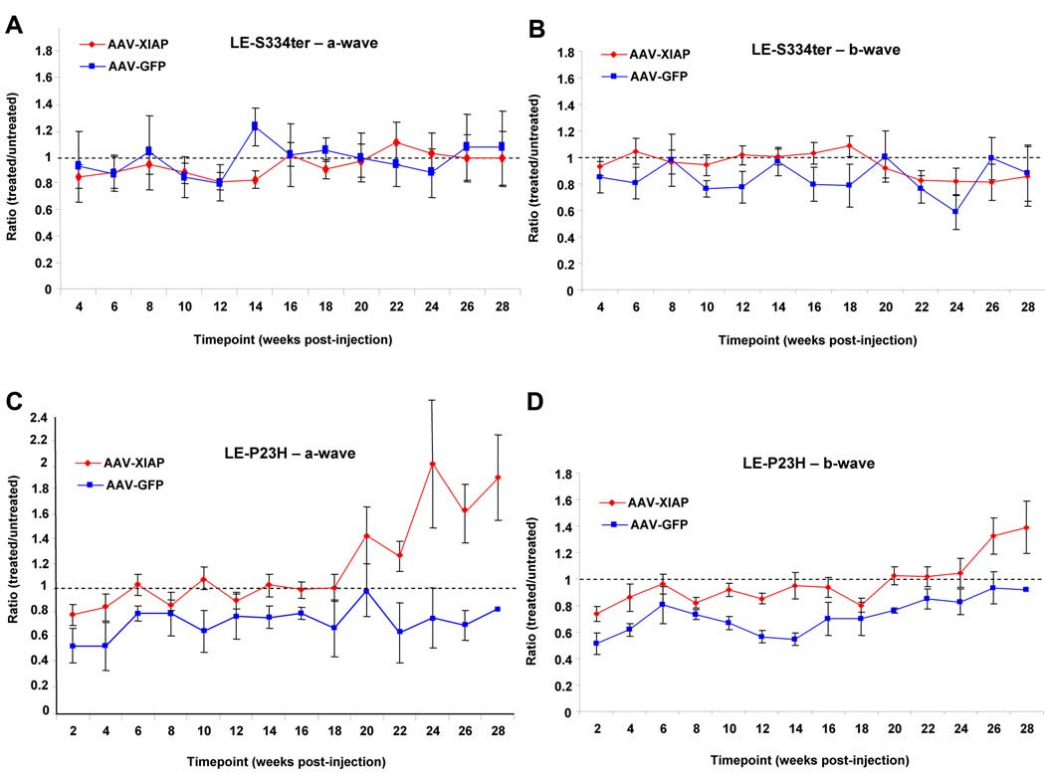
To assess functional preservation, the a- or b-wave amplitude of the treated eye was divided by that of the contralateral, untreated eye to give an amplitude ratio. Amplitude ratios from the 8^th^ luminance step (0.63 cd.s/m^2^) are graphed. Amplitude ratios for LE-S334ter animals (A,B) show no significant differences between treated and control retinas. Ratios for LE-P23H animals (C,D) show significant differences for a-wave (p<0.001) and b-wave (p<0.03) by ANOVA. For statistical analyses, N values were 12 and 3 for XIAP and GFP respectively.

For the S334ter animals, no preservation of the a-wave was identified at any of the timepoints (Fig. 4A). The b-wave amplitude ratios for XIAP treated eyes appeared to be somewhat, although not significantly, better than the GFP control ratios up to approximately 18 weeks post-injection (Fig. 4B). Thereafter, the performance in the XIAP group declined and was comparable to the GFP control group. Overall, there was no lasting or significant functional protection resulting from XIAP treatment in the S334ter animals.

For the P23H animals, there was a clear difference between the a-wave and b-wave amplitude ratios for the XIAP treated group in comparison to the GFP treated group across all timepoints (Fig. 4C, D). These differences appeared to increase with time, as the degeneration progressed in the contralateral untreated eye. A repeated measures ANOVA with Bonferroni-Dunn correction revealed a significant difference between the XIAP and GFP treated groups for both the a-wave (p<0.001) and b-wave amplitudes (P<0.03).

## Discussion

A comprehensive gene therapy approach which would target retinal degeneration must be widely applicable given the number of different mutations which can cause retinal disease. In RP alone, more than 39 different genetic loci have been discovered and the causative gene has only been identified in 28 of these [4]. Given all the different mutations in all the different genes, gene therapy for RP would be a daunting task if one attempted to target all possible mutations. A final common pathway for all the types of RP is cell death by apoptosis. Thus, targeting and preventing the apoptotic event may represent a general and pragmatic approach to the treatment of a wide variety of mutations leading to disease.

There is controversy in the literature on the mechanism of apoptotic death in retinal degeneration with regards to the presence [35], [36], [37] or absence [38] of caspase activation. The success of our gene therapy in the two animal models presented in this study suggests that caspases are involved, since XIAP binds to and inhibits the action of caspases 3, 7 and 9. Our results show great promise for XIAP gene therapy in the treatment of RP. We show strong preservation of ONL morphology coincident with XIAP over-expression in both P23H and S334ter rats, while non-injected controls and AAV-GFP-treated animals undergo progressive degeneration. ERG data reveal significant differences in retinal function in the eyes of XIAP-treated P23H animals in comparison to those treated with GFP for both a-wave and b-wave measures. These differences are not as pronounced in the early stages of disease progression as would be predicted based on the loss of photoreceptors, most likely due to some level of redundancy in the retinal circuitry. However, ERG differences between XIAP and control groups appear to increase with time, undoubtedly due to the continuing retinal degeneration in the control eyes. The finding of a greater preservation of a-wave amplitude in comparison to b-wave (indicated by larger amplitude ratios when comparing treated to untreated eyes) is expected. This is based on the fact that the a-wave is generated primarily by the photoreceptors, which were the targets of the gene therapy, whereas the b-wave is indicative of overall retinal function.

While significant differences in the ERG amplitude are evident between XIAP and GFP-treated P23H animals, several factors likely masked the true magnitude of the protective effect. These variables also explain why the amplitude ratios were slightly below 1 at the earlier timepoints, even for the XIAP-treated group. The trans-corneal subretinal injection procedure may cause some damage to the eye that might impede ERG recordings. The nature of this damage could be wide-ranging. The procedure causes a localized detachment of the retina, which eventually resolves but may leave some residual damage. The injection often causes some bleeding of pigment into the corneal scar, that in a few occasions can lead to completely ‘bound’ pupils (synechiae) which have lost the ability to dilate. In addition, the trans-corneal procedure has been estimated to cause cataracts in 20–40% of animals [39] . All of these abnormalities can obstruct the transmission of the ERG light stimulus into the eye. In this study, animals with completely bound pupils or large cataracts were removed from the study, but those with corneal bleeds or minor cataracts remained, possibly reducing the amplitude of the ERG response in the treated eyes in comparison to the contralateral untreated eyes. An additional factor which may have masked the magnitude of the protective effect is the fact that a single subretinal injection covers approximately 20% of the retina [40], but the ERG records the response from the whole retina. Thus, the full extent of functional protection at the site of injection could be masked by the response of the majority of the retina which was not covered by the virus and continued to degenerate. Many of these limitations would not be relevant to the treatment of human disease which would involve much more sophisticated surgical procedures, viral delivery and multi-focal ERG assessment.

The completely different nature of the mutations in the two transgenic lines may offer an explanation for the presence of long-term functional protection in P23H animals and the clear absence of such protection in the S334ter line. Rhodopsin mutations have been divided into several classes based predominantly on their behavior in tissue culture experiments [41], [42]. S334ter represents a Class I mutant, while P23H is a member of the more predominant Class II mutations. Class I mutants have impaired trafficking to the rod outer segment, where phototransduction occurs. The S334ter mutant protein accumulates to high levels in the plasma membrane, where it may interfere with synaptic transmission, or the transport of cellular cargo [11], [12]. Class II mutants such as P23H are thought to be defective in protein folding, and prone to aggregation (reviewed in [41], [42]). P23H mutant protein becomes multi-ubiquitinylated, and accumulates in cytoplasmic aggresomes; this destabilizes the protein and targets it for degradation via the ubiquitin proteosome system [43], [44]. The precise mechanisms by which these mutations lead to apoptotic cell death remains unknown, but they clearly have very different effects on the transport and function of rhodopsin.

The P23H results are more relevant for the treatment of human disease, since this same mutation is causative in approximately 12% of human adRP cases in North America [10]. Since P23H also represents the larger group of class II rhodopsin mutations, XIAP gene therapy may be equally effective in protecting against other RP-causing misfolding rhodopsin mutations. Even if XIAP gene therapy only protects the structure (and not the function) of the rod photoreceptors, this may still have significant effects on visual outcome in humans. In RP, the initial phase of mutant rod cell loss is followed by a wave of cone cell death [45]–[48]. The underlying mechanisms of these secondary effects are not known, but it may be that apoptosis of the rods causes changes in chemical or mechanical forces on the surviving cones, resulting from the release of toxic factors into the retina or the loss of structural support. By preventing rod structural loss, XIAP may still help to retain cone structure and function (i.e. day and colour vision). This would be very important in humans, who have a cone-dominant central retina.

Previous gene therapy studies for the treatment of retinal degenerations have targeted the apoptotic response. These studies have examined the therapeutic potential of over-expression of the bcl-2 family of proteins through viral delivery or transgenesis, but the success of these studies has been limited. Bcl-2 is able to delay photoreceptor degeneration in various murine disease models, but the protective effects are transient, persisting for only 4 to 6 weeks before regressing to the untreated levels [49]–[51]. Moreover, high levels of bcl-2 over-expression in photoreceptors promote retinal degeneration rather than preventing it, and so gene dosage is critical [50]. In addition, neuron specific enolase (NSE)-driven bcl-2 over-expression prevents the naturally occurring apoptotic mechanisms required during normal development [52]. In our experience, increasing levels of XIAP have not had any deleterious effects on photoreceptors. Furthermore, XIAP transgenic animals, where the transgene is driven by the NSE promoter, undergo normal developmental apoptosis [27].

These results clearly demonstrate the potential of XIAP gene therapy for the treatment of inherited retinal degenerations. It is important to note that the XIAP treatment does not represent an all-encompassing gene therapy strategy for retinal degeneration. Some forms of retinal disease may be associated with developmental, structural or RPE defects. In these cases, preventing cell death of photoreceptors will not repair the damage and will not induce vision where none exists. However, many retinal degenerations are associated with normal vision and development early in life and are later triggered by some, as yet unknown, mechanism which results in photoreceptor death by apoptosis. Thus, targeting apoptosis through XIAP gene therapy may yield a general and widely applicable approach for these types of retinal disease.

## Materials and Methods

### Animals

Line 1 P23H and line 4 S334ter transgenic rats were generously donated by Dr. Matt LaVail (UCSF, CA). Homozygous transgenic animals were crossed with wild-type Long Evans rats (Charles River Laboratories, Wilmington, MA), to generate transgenic pigmented offspring. Animals were reared under standard laboratory conditions in accordance with the Association of Research in Vision and Ophthalmology (ARVO) Statement for the Use of Animals in Ophthalmic and Vision Research and the guidelines of the University of Ottawa Animal Care and Veterinary Service.

### Construction of rAAV Vectors

A cDNA construct encoding the full-length human XIAP open reading frame with an N-terminal hemagglutinin (HA) tag was inserted into a pTR vector under the control of the chicken β-actin promoter. A GFP construct was generated in a similar manner for use as a negative control. A woodchuck hepatitis virus post-transcriptional regulatory element (WPRE) was placed in the 3′ untranslated region of the construct to enhance expression of the viral transgene. Serotype 5 rAAV was generated as previously described [53], [54]. The viral titers were adjusted to 4.0×10^13 ^physical particles/ml for AAV-GFP, and 3.0×10^14^ physical particles/ml for AAV-XIAP. Ratios of physical to infectious particles were lower than 100. The rAAV vectors, purified using iodixanol gradient/heparin-affinity chromatography, were more than 99% pure, as judged by polyacrylamide silver-stained gel electrophoresis (data not shown). Contaminating helper adenovirus and wild-type AAV, assayed by serial dilution cytopathic effect or infectious center assay respectively, were below detection levels.

### Subretinal Injections

Subretinal injections were done within 3 days of the opening of the eyes, between post-natal day 14 (P14) and P17. Injections were conducted under isofluorane anesthesia, as previously described [33], [55]. A 2-*u*l volume of virus (rAAV-XIAP or rAAV-GFP), diluted 50:1 with fluorescein tracer was injected into the sub-retinal space of the eye, in the central retina, nasal to the optic disc. The fluorescein tracer allowed immediate visualization and evaluation of the injection location. The sub-retinal injection procedure caused a localized retinal detachment which has been previously shown to resolve within several days post surgery [55]. For the S334ter studies, 20 animals received rAAV-XIAP and 10 animals received rAAV-GFP. Two animals were eliminated from each group due to cataracts or complete synechiae, resulting in final numbers of 18 for rAAV-XIAP and 8 for rAAV-GFP. For the P23H studies, 20 animals were injected with rAAV-XIAP and 7 received rAAV-GFP. Two animals were eliminated from the first group and 3 from the second, resulting in 18 animals for rAAV-XIAP and 4 animals for rAAV-GFP. The injected eye was alternated between right and left, to eliminate any potential bias due to slight differences in the corneal surface electrodes used for ERG recordings.

### Electroretinography

Full-field scotopic/photopic electroretinograms were generated using the ESPION system (Diagnosys LLC, Littleton, MA). Rats were weighed and dark-adapted overnight prior to ERG analysis. Under safe-light conditions, the animals were anaesthetized with an intraperitoneal injection of 30mg/kg ketamine hydrochloride (Bimeda-MTC, Cambridge, ON) and 0.5 mg/kg medetomidine hydrochloride (Domitor, Novartis, Finland). After 1 hour under anaesthesia, the medetomidine was reversed using 1 mg/kg of atipamezole hydrochloride (Antisedan, Novartis, Finland). Eyes were dilated using both 1% tropicamide and 2.5% phenylephrine hydrochloride (Alcon Canada), applied at 20 and 5 minutes prior to the test, in order to ensure complete dilation. During testing, animals were placed on a water circulating heating pad (Gaymar T/Pump, Orchard Park, NY) to maintain a constant body temperature throughout the test.

Silver wire loop electrodes were placed on both corneas with a drop of 0.3% hypromellose (Novartis) to maintain corneal hydration. A gold minidisc reference electrode was placed on the tongue and a ground needle electrode was placed subcutaneously in the tail. The animal's head was positioned under the center of the Ganzfeld dome. Single flash stimuli (4 ms duration) were presented at eleven increasing intensities ranging from 0.001 cd.s/m^2^ to 25 cd.s/m^2^. Five ERG traces were obtained and averaged for each luminance step. ERGs were recorded for all animals at biweekly timepoints, commencing two weeks after the subretinal injection procedure, and continuing up to 28 weeks post-injection. The analyses of ERG data presented here were conducted on the 8^th^ luminance step (0.63 cd.s/m^2^) of the 11 step series.

### Western Analysis

Western blots were generated with protein extracts from XIAP injected eyes and contralateral uninjected control eyes. Eyes were enucleated and retinas isolated two weeks after the subretinal injection. Each retina was sonicated in 200 µL RIPA buffer (50 mM Tris-HCl [pH 7.4], 150 mM NaCl, 1 mM EDTA, 1% NP-40, 0.25% Na-deoxycholate, 1mM NaF, 1mM Na_3_VO_4_, 1 mM phenylmethylsulfonyl fluoride [PMSF], and 10 µg/mL each of leupeptin and aprotinin). The samples were rocked at 4°C for 1 hour, and then centrifuged in an IEC microfuge for 15 minutes at 4°C and 13000 RPM. Protein concentration was determined using the DC protein assay (Bio-Rad). Twenty micrograms of protein from each eye was electrophoresed on a 12% sodium dodecyl sulfate-polyacrylamide (SDS-PAGE) gel, and transferred onto a polyvinylidene fluoride (PVDF) membrane (Immobilon P, Millipore). Blots were probed with an antibody to the HA epitope tag (1∶ 2,500 Rat anti-HA, Roche Molecular Biochemicals), followed by an anti-Rat horseradish peroxidase secondary antibody (1 in 20,000, Amersham Pharmacia). Protein detection was achieved using a SuperSignal West Pico Chemiluminescent Substrate detection kit (Pierce Biotechnology, Rockford, IL).

### Tissue Fixation and Processing

Eyes were sampled at 28 weeks post-injection, (at approx. 30 weeks of age in the animal), when the degeneration in the untreated, control eye was extensive. Tissue sampling was conducted as previously described [25]. Tissue sections (12–15 µm) were cut using a Shandon cryostat, air dried for 2 hours, and stored at −20°C with dessicant. Haematoxylin & Eosin (H & E) staining was done according to standard protocols.

Measurements of ONL thickness were taken near the site of the injection in the treated animals and at the same distance from the optic nerve head in the contralateral untreated eyes. Images were obtained at 40× magnification using a Zeiss Axioskop microscope with an AxioCam Hrc camera. ONL thickness was measured in five evenly spaced sections near the injection site for each retinal image, averaged and compared between treated eyes and their contralateral, untreated counterparts.

### Immunohistochemistry

Immunohistochemistry was used to localize the HA tagged XIAP transgene. Sections were probed with a rat anti-HA primary antibody (1∶500, Roche Molecular Biochemicals) followed a goat anti-rat IgG secondary antibody (Alexafluor 488, 1∶400, Molecular Probes). Slides were counterstained with the nuclear stain 4′, 6′-diamindino-2-phenylindole dihydrochloride (DAPI; 1∶10000, D9542, Sigma-Aldrich). GFP fluorescence was readily observable under fluorescence filters on the microscope after re-hydration of retinal sections. Images were obtained using a Zeiss Axioskop light microscope with a Zeiss AxioCam HRc camera.

### Statistical Analysis

For assessment of ONL thickness, a 1-tailed paired t-test was used for comparisons between injected and uninjected eyes within the same animal, while a 1-tailed independent samples t-test was used to detect differences between the XIAP treated eyes and GFP injected control eyes in different groups of animals.

For ERG analyses, a repeated measures ANOVA with a Bonferroni-Dunn correction for multiple comparisons was used to detect a difference between XIAP and GFP treated groups. ANOVA requires equal numbers of data points at all timepoints; thus only animals which had ERG recordings every two weeks and were present in the study until 28 weeks post-injection were used for these analyses. Animals that lacked ERG data at any timepoint because of difficulties with plane of anesthesia were not included in the analysis. All statistics were done using StatView Version 4.57 (Abacus Concepts, Berkeley, CA, USA).
